# Power analysis of longitudinal studies with piecewise linear growth and attrition

**DOI:** 10.3758/s13428-022-01791-x

**Published:** 2022-02-07

**Authors:** Mirjam Moerbeek

**Affiliations:** Department of Methodology and Statistics, Utrecht University, PO Box 80140, 3508 TC Utrecht, the Netherlands

**Keywords:** Multilevel model, Power, Piecewise growth model, Multiphase, Shiny app

## Abstract

In longitudinal research, the development of some outcome variable(s) over time (or age) is studied. Such relations are not necessarily smooth, and piecewise growth models may be used to account for differential growth rates before and after a turning point in time. Such models have been well developed, but the literature on power analysis for these models is scarce. This study investigates the power needed to detect differential growth for linear–linear piecewise growth models in further detail while taking into account the possibility of attrition. Attrition is modeled using the Weibull survival function, which allows for increasing, decreasing or constant attrition across time. Furthermore, this work takes into account the realistic situation where subjects do not necessarily have the same turning point. A multilevel mixed model is used to model the relation between time and outcome, and to derive the relation between sample size and power. The required sample size to achieve a desired power is smallest when the turning points are located halfway through the study and when all subjects have the same turning point. Attrition has a diminishing effect on power, especially when the probability of attrition is largest at the beginning of the study. An example on alcohol use during middle and high school shows how to perform a power analysis. The methodology has been implemented in a Shiny app to facilitate power calculations for future studies.

**Supplementary Information:**

The online version contains supplementary material available at 10.3758/s13428-022-01791-x.

## Introduction

In the social and behavioral sciences, subjects are often measured at multiple points across time or age in order to study changes in abilities, behavior, opinion, attitude and so forth. Data that arise from such longitudinal studies are often analyzed by means of a multilevel model (Goldstein, 2011; Hox et al., 2018; Raudenbush & Bryk, 2002) or a latent growth curve model (Duncan et al., 2006).

Most often, smooth growth trajectories, such as those modeled by linear, polynomial and nonlinear (e.g., exponential) relations between time (or age) and response, are fitted. However, some longitudinal studies may show non-smooth patterns of change. Growth may show a sharp change after the occurrence of some important life event, such as first criminal offense, entry into parenthood, retirement, or death of spouse. One example is a study on the change in alcohol use across middle and high school (Li et al., 2001). The authors found a higher linear growth rate in high school as compared to middle school. Discontinuities in growth may also be observed in experimental studies where the beginning or end of an intervention is the turning point. An example is a study on the change in bulimia severity, depression and self-concept of female patients during and after treatment with guided self-change treatment or cognitive behavioral therapy. Cognitive behavioral therapy showed greater improvement during therapy, while guided self-change treatment showed more continued improvement post-treatment. Data obtained from such studies are known as interrupted time-series data and can be analyzed by means of multilevel or latent growth curve models (Duncan et al., 2004; Flora, 2008; Grimm & Marcoulides, 2016; Harring et al., 2021; Muggeo et al., 2014) using one or more turning points to distinguish different phases across time and by specifying differential growth rates across these phases.

Longitudinal research often requires considerable effort, money and time from both the researchers and the participants. It is therefore important that a longitudinal study is designed carefully. Among other components, the number of subjects, number of measurements per subject and the study duration must be decided upon, and it has to be determined whether sufficient statistical power can be achieved. Over the past three decades, a few dozen papers on the design of longitudinal studies have appeared (e.g., De Jong et al., 2010; Fan, 2003; Galbraith & Marschner, 2002; Hedeker et al., 1999; Moerbeek, 2008, 2011; Raudenbush & Liu, 2001; Zhang & Wang, 2009) The focus of these papers is on smooth growth trajectories with linear or polynomial growth. This implies that these methods cannot be used for piecewise growth models, as a power analysis for a certain model cannot be done on the basis of another model. Moreover, design questions for a piecewise growth model are different from those of a polynomial growth model. For instance, power may be determined not only by the number of measurements per subject, but also by the number of measurements per phase. In addition, the model parameter for which a power analysis is to be done depends on what type of model is used. In longitudinal studies with smooth growth trajectories, it is the growth rate across time and/or the interaction of the growth rate with another variable, such as treatment condition. In piecewise growth models it is the change in growth from one phase to the next.

Sample size guidelines for piecewise growth models are scarce; to the author’s knowledge there exist only two relevant papers. Diallo and Morin (2015) conducted a simulation study with 6, 8 and 10 measurements and a turning point at time point 2, 3 or 4. They showed that power increases with increasing sample size, number of measurements, the difference between the two slopes and the correlation between the two slopes. Larger power was observed when the turning point was at the third or fourth time point than at the second time point. Power decreased when the variance of the second slope increased. Segalas et al. (2019) also conducted a simulation study; they used study duration of 21. Each subject was allowed to have their own turning point, and attrition was assumed to be absent or to occur at a constant rate. Larger power was observed for a larger slope difference, a larger sample size and when attrition was absent. Power was larger when the turning point was located at time point 15 than at time point 10, and when the variability in turning points was lower.

Although these two studies are very useful, they also have their limitations. Diallo and Morin restricted their work to scenarios in which each subject had the same turning point, which may not always be realistic. For instance, the age at which subjects graduate from college or enter parenthood varies across subjects. They also ignored the possibility of attrition, while in longitudinal studies attrition is the rule rather than the exception. Segalas and coauthors did take into account the variability in turning points and the possibility of attrition. However, they assumed constant attrition rates across time, while attrition rates may very well vary across time. Furthermore, both papers based their power calculations on simulations, which may be time-consuming and require specific software (in their case SAS and Mplus, which are not free of charge).

The aim of this contribution is to further investigate the power to detect a difference in growth rates in piecewise linear–linear growth models. The study investigates how power is influenced by the location of turning points; in other words, is the highest power achieved when most subjects have a turning point at the beginning, halfway or at the end of the study? Furthermore, it investigates whether higher power is achieved when all subjects have the same turning point or when they have different turning points. In addition, the loss in efficiency due to attrition is studied. Attrition is modeled using the Weibull survival function, which allows for increasing, decreasing and constant attrition rates during the course of the study. The methodology has been implemented in a Shiny app to facilitate power analysis for future studies.

The remainder of this paper is organized as follows. In the next section the multilevel mixed model for piecewise growth is presented. In the following section it is shown how power to detect differential growth is calculated. This section also introduces the Shiny app. The section thereafter shows how power is influenced by the location of and variability in turning points in studies without attrition. The following section quantifies the loss in efficiency due to attrition. The final section presents conclusions and a discussion, with directions for future research.

## Multilevel mixed model

Repeated measurements across time are nested within subjects; hence the data have a multilevel structure and can be analyzed using the multilevel mixed model (Goldstein, 2011; Hox et al., 2018; Snijders & Bosker, 2012), which is also known as the hierarchical (linear) model (Raudenbush & Bryk, 2002). An alternative model is the latent growth curve model within the general framework of structural equation models (Duncan et al., 2006; Flora, 2008).

The duration of the study is denoted as *D*. The aim is to measure each subject *i* = 1, …, *n* at equidistant time points *t* = 0, 1, 2, …, *D*. As a measurement is also taken at baseline (*t* = 0), the aim is to measure each subject at *m* = *D* + 1 time points. However, subjects may prematurely drop out of the study, meaning that the number of measurements may vary across subjects. The number of measurements for subject *i* is denoted as *m*_*i*_.

The study is split in two different time phases, with phase 1 beginning at time point *t* = 0 and phase 2 beginning at time point *t* = *T*_*i*_. The latter time point is the turning point, which may be either constant or varying across subjects. When all subjects have the same turning point (i.e., *T*_*i*_ = *T* ∀ *i*), the turning point cannot be located at *t* = 0 or *t* = *D* because that would mean the study has only one phase.

In both phases a linear relation between time and response is assumed. The multilevel mixed model for subject *i* at time point *t* is then given by

1 $${y}_{ti}={\pi}_{0i}+{\pi}_{1i}{t}_{1 ti}+{\pi}_{2i}{t}_{2 ti}+{e}_{ti}.$$

The variables *t*_1*ti*_ and *t*_2*ti*_ are time indicators for the first and second phase of the study for subject *i*. They are coded as follows:

2 $${\displaystyle \begin{array}{c}{t}_{1 ti}=t\kern0.5em \mathrm{if}\ t\le {T}_i\kern0.5em \mathrm{and}\ {t}_{1 ti}={T}_i\kern0.75em \mathrm{if}\kern0.5em t>{T}_i,\\ {}{t}_{2 ti}=0\kern0.5em \mathrm{if}\kern0.5em t\le {T}_i\kern0.5em \mathrm{and}\kern0.5em {t}_{2 ti}=t-{T}_i\kern0.5em \mathrm{if}\kern0.5em t>{T}_i\end{array}}$$

Consider as an example subject *i* with *m*_*i*_ = 7 time points and a turning point *T*_*i*_ = 3. The design matrix for this subject is given by

3 $${\boldsymbol{X}}_i=\left(\begin{array}{ccc}1& 0& 0\\ {}1& 1& 0\\ {}1& 2& 0\\ {}1& 3& 0\\ {}1& 3& 1\\ {}1& 3& 2\\ {}1& 3& 3\end{array}\right).$$

The associated regression weights *π*_0*i*_, *π*_1*i*_ and *π*_2*i*_ are the baseline score and growth rates in phase 1 and 2, respectively. Each of them is assumed to randomly vary across subjects:

4 $${\displaystyle \begin{array}{c}{\pi}_{0i}={\beta}_0+{u}_{0i},\\ {}\begin{array}{c}{\pi}_{1i}={\beta}_1+{u}_{1i},\\ {}{\pi}_{2i}={\beta}_2+{u}_{2i}.\end{array}\end{array}}$$

Here, the regression weights *β*_0_, *β*_1_ and *β*_2_ are the average intercept and growth rates, and the random variables *u*_0*i*_, *u*_1*i*_ and *u*_2*i*_ are the deviations of subject *i* from these averages. As each of the three regression coefficients *π*_0*i*_, *π*_1*i*_ and *π*_2*i*_ has a random effect, the design matrix ***Z***_*i*_ for the random part is equal to the design matrix ***X***_*i*_ for the fixed part.

The random variables *u*_0*i*_, *u*_1*i*_ and *u*_2*i*_ are assumed to follow a multivariate normal distribution with means equal to zero and covariance matrix

5 $$\mathit{\operatorname{cov}}\left({\boldsymbol{u}}_i\right)=\mathit{\operatorname{cov}}\left(\begin{array}{c}{u}_{0i}\\ {}{u}_{1i}\\ {}{u}_{2i}\end{array}\right)=\left(\begin{array}{ccc}{\sigma}_{u0}^2& {\sigma}_{01}& {\sigma}_{02}\\ {}{\sigma}_{01}& {\sigma}_{u1}^2& {\sigma}_{12}\\ {}{\sigma}_{02}& {\sigma}_{12}& {\sigma}_{u2}^2\end{array}\right).$$

These random variables are assumed to be independent from the residuals *e*_*i*0_, *e*_*i*2_, …, *e*_*iD*_. These residuals are assumed to follow a multivariate normal distribution with means equal to zero and covariance matrix ${\sigma}_e^2{\boldsymbol{I}}_i$, where ***I***_*i*_ is the (*m*_*i*_ + 1) × (*m*_*i*_ + 1) identity matrix and ${\sigma}_e^2$ is the variance of the residual term *e*_*ti*_.

The model for subject *i* can be written in matrix notation:

6 $${\boldsymbol{y}}_i={\boldsymbol{X}}_i\boldsymbol{\beta} +{\boldsymbol{Z}}_i{\boldsymbol{u}}_i+{\boldsymbol{e}}_i$$

with ***y***_*i*_ the vector of responses, ***X***_*i*_ the design matrix for the fixed part, ***β*** = (*β*_0_, *β*_1_, *β*_2_)′ the vector of regression weights, ***Z***_*i*_ the design matrix for the random part, ***u***_*i*_ = (*u*_0*i*_, *u*_1*i*_, *u*_2*i*_)′ the vector of random variables and ***e***_*i*_ = (*e*_*i*1_, *e*_*i*2_, …, *e*_*i*(*m* + 1)_)′ the vector of residuals.

Given the covariance matrices for the random effects, the covariance matrix (conditional on the fixed effects) of the responses of subject *i* is

7 $$\mathit{\operatorname{cov}}\left({\boldsymbol{y}}_i|\ {\boldsymbol{X}}_i\boldsymbol{\beta} \right)={\boldsymbol{V}}_i={\boldsymbol{Z}}_i\mathit{\operatorname{cov}}\left({\boldsymbol{u}}_i\right){{\boldsymbol{Z}}_i}^{\prime }+{\sigma}_e^2{\boldsymbol{I}}_i.$$

Once the variances and covariances in *cov*(***u***_*i*_) and the variance ${\sigma}_e^2$ have been estimated, they can be plugged into the equation above to get ${\hat{\boldsymbol{V}}}_i$. The vector of regression coefficient is then estimated by

8 $$\hat{\boldsymbol{\beta}}={\left(\sum \nolimits_{i=1}^n{\boldsymbol{X}}_i^{\prime} {\left({\hat{\boldsymbol{V}}}_i\right)}^{-1}{\boldsymbol{X}}_i\right)}^{-1}\sum \nolimits_{i=1}^n{\boldsymbol{X}}_i^{\prime}{\left({\hat{\boldsymbol{V}}}_i\right)}^{-1}{\boldsymbol{y}}_i.$$

This is the maximum likelihood estimator of fixed effects of the linear mixed effects model in equation (6). The associated covariance matrix is estimated as

9 $$\hat{\mathit{\operatorname{cov}}}\left(\hat{\boldsymbol{\beta}}\right)=\left(\begin{array}{ccc}\hat{\mathit{\operatorname{var}}}\left({\hat{\beta}}_0\right)& \hat{\mathit{\operatorname{cov}}}\left({\hat{\beta}}_0,{\hat{\beta}}_1\right)& \hat{\mathit{\operatorname{cov}}}\left({\hat{\beta}}_0,{\hat{\beta}}_2\right)\\ {}\hat{\mathit{\operatorname{cov}}}\left({\hat{\beta}}_0,{\hat{\beta}}_1\right)& \hat{\mathit{\operatorname{var}}}\left({\hat{\beta}}_1\right)& \hat{\mathit{\operatorname{cov}}}\left({\hat{\beta}}_1,{\hat{\beta}}_2\right)\\ {}\hat{\mathit{\operatorname{cov}}}\left({\hat{\beta}}_0,{\hat{\beta}}_2\right)& \hat{\mathit{\operatorname{cov}}}\left({\hat{\beta}}_1,{\hat{\beta}}_2\right)& \hat{\mathit{\operatorname{var}}}\left({\hat{\beta}}_2\right)\end{array}\right)={\left(\sum \nolimits_{i=1}^n{\boldsymbol{X}}_i^{\prime} {\left({\hat{\boldsymbol{V}}}_i\right)}^{-1}{\boldsymbol{X}}_i\right)}^{-1}.$$

The variances $\hat{\mathit{\operatorname{var}}}\left({\hat{\beta}}_1\right)$ and $\hat{\mathit{\operatorname{var}}}\left({\hat{\beta}}_2\right)$ and covariance $\hat{\mathit{\operatorname{cov}}}\left({\hat{\beta}}_1,{\hat{\beta}}_2\right)$ are used to study the relation between sample size and power to detect a difference in growth rates across the two phases.

## Statistical power to detect differential growth

The main question is whether the growth rates in the two phases are equal to one another. The corresponding null hypothesis is *H*_0_ : *β*_1_ = *β*_2_, which can also be formulated as *H*_0_ : *β*_1_ − *β*_2_ = 0. This difference is estimated by plugging in the estimates of the regression coefficients, and the associated variance is estimated as $\hat{\mathit{\operatorname{var}}}\left({\hat{\beta}}_1-{\hat{\beta}}_2\right)=\hat{\mathit{\operatorname{var}}}\left({\hat{\beta}}_1\right)+\hat{\mathit{\operatorname{var}}}\left({\hat{\beta}}_2\right)-\hat{2\mathit{\operatorname{cov}}}\left({\hat{\beta}}_1,{\hat{\beta}}_2\right)$. The variances and covariance at the right side of this equation follow from the covariance matrix (9).

If there indeed exists a difference in growth rates across the two phases in the population, then one would like to detect it with sufficient statistical power. The relation between the difference in growth rates *β*_1_ − *β*_2_, the variance *var*(*β*_1_ − *β*_2_), statistical power 1 − *β*, and type I error rate *α* is given by

10 $$\frac{\beta_1-{\beta}_2}{\sqrt{\mathit{\operatorname{var}}\left({\beta}_1-{\beta}_2\right)}}={z}_{1-\alpha }+{z}_{1-\beta }.$$

This relation holds for a one-sided alternative *H*_1_ : *β*_1_ − *β*_2_ ***>*** 0 or *H*_0_ : *β*_1_ − *β*_2_ ***<*** 0; for two-sided alternative *H*_0_ : *β*_1_ − *β*_2_ ≠ 0, *z*_1 − *α*_ is replaced by *z*_1 − *α*/2_.

In the design phase of a study, the difference in means is often not known. This causes a vicious cycle: the study is to be conducted to gain insight into the difference in growth rates, but to design the study such that it has sufficient power, the population value of the difference in growth rates needs to be known in advance. To escape the vicious cycle, one can consult the literature for similar studies in the past to gain insight into plausible values for the difference in growth rates.

The variance *var*(*β*_1_ − *β*_2_) depends on the number of subjects, the number of measurements per subject and the location of the turning point in the case where all subjects have the same turning point. In the case of varying turning points, the variance depends on the distribution of turning points. Furthermore, this variance also depends on the rate of attrition across the study. In addition, it is a function of the variance and covariance components in Eq. (5) and the variance ${\sigma}_e^2$. The expression for the variance *var*(*β*_1_ − *β*_2_) cannot be captured by a simple mathematical expression. For that reason, matrix algebra should be used to calculate the value of the variance for each specific study at hand. The online 14 shows the results of a small simulation study. The power as calculated using matrix algebra is almost the same as that obtained from simulation.

### Shiny app

To facilitate the use of the methodology presented herein, a Shiny app was developed to study the relation between number of subjects and power. First, the user has to specify the duration of the study and the distribution of turning points: the proportion of subjects that have a turning point at each of the time points *t* = 0, 1, …, *D*. Second, the values of all variance and covariance components have to be specified, along with the residual variance. Third, the population values of the growth rates in both phases have to be specified, along with the type I error rate and whether a one- or two-sided alternative is used. Finally, the parameters *ω* and *γ* of the Weibul attrition function have to be specified (see later in this contribution for an explanation of these parameters). Once all parameters have been specified, the app shows the relation between number of subjects and power for the growth rates in phases 1 and 2 and for the difference in growth rates. Power levels are shown for the user-selected degree of attrition and for zero attrition. By hovering over a graph, the power level for a selected number of subjects is displayed. The app can be found online at https://utrecht-university.shinyapps.io/Power_Piecewise_Growth/

## Designing studies without attrition

The aim of this section is to study how the distribution of turning points affects the sample size to achieve a power 1 − *β* = 0.8 to detect a slope difference in turning points in a two-sided test with type I error rate *α* = 0.05 in a study without attrition.

### Parameter values

Statistical power depends on the values of the model parameters. The population values of the regression coefficients, variance and covariance components are taken from Diallo and Morin (2015); a rationale for these values can be found in that paper.

**Fig. 1:**
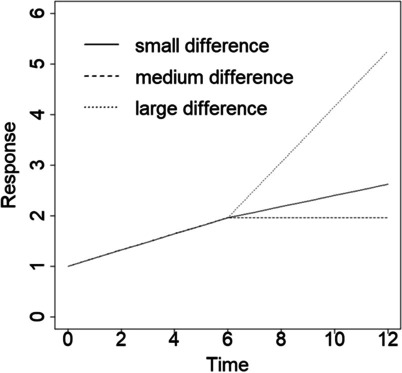
Growth curve for mean growth rate equal to *β*_1_ = 0.16 in phase 1 and three mean growth rates *β*_2_ in phase 2 (resulting in three differences in growth rates)

The average response at *t* = 0 is *β*_0_ = 1. The mean growth rate in the first phase is *β*_1_ = 0.16, while the average growth rate in the second phase is *β*_2_ = 0.11, 0.0 or 0.55. Given these values, the difference in growth rates is *β*_1_ − *β*_2_ = 0.05, *β*_1_ − *β*_2_ = 0.16 or *β*_1_ − *β*_2_ = 0.39, respectively. The mean growth curves in the first and second phase of the study are presented in Fig. 1.

The variance component for the random intercept is *var*(*u*_0*j*_) = 0.2, the variance component for the growth rate in the first phase is *var*(*u*_1*j*_) = 0.1, and the variance component for the growth rate in the second phase is *var*(*u*_2*j*_) = 0.16. The correlation between the random intercept and phase 1 slope is *cor*(*u*_0*j*_, *u*_1*j*_) = 0.1, the correlation between the random intercept and phase 2 slope is *cor*(*u*_0*j*_, *u*_2*j*_) = 0, and the correlation between the two random slopes is *cor*(*u*_1*j*_, *u*_2*j*_) = 0. Finally, the residual variance is *var*(*e*_*ij*_) = 0.2 and does not vary across the time points.

### Distribution of turning points

**Fig. 2:**
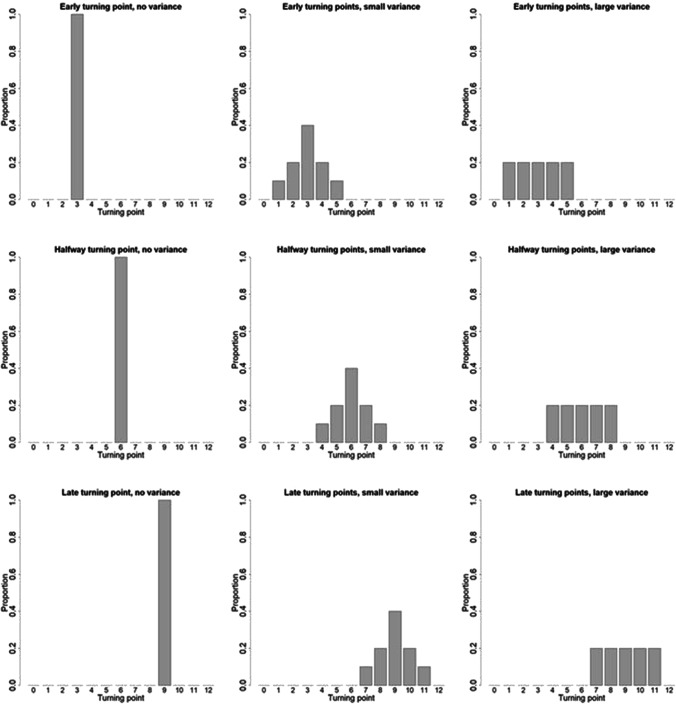
Distribution of turning points in a study with duration *D* = 12

Figure 2 gives the nine distributions of turning points *T*_*i*_ that will be used in this section and the next. The bars in this figure show the proportion of subjects that have a turning point at time points *t* = 0, 1, …, 12 in a study with duration *D* = 12. In the top row the turning points are located at the beginning of the study, with a mean *μ*_*T*_ = 3; in the middle row they are located halfway through the course of the study (*μ*_*T*_ = 6) and in the bottom row at the end of the study (*μ*_*T*_ = 9). In the left column all subjects have the same turning point, meaning the variance in turning points ${\sigma}_T^2$ is zero. In the middle column there is a small variance in turning points (${\sigma}_T^2=1.33$), while in the right column there is large variance (${\sigma}_T^2=2.22$)

### Results

Table 1 shows the required sample size to achieve a power 1 − *β* = 0.8 to detect a difference between the two slopes as a function of the distribution of turning points and for three differences between the two slopes (two-sided test with *α* = 0.05). As is obvious, smaller sample sizes are needed for larger slope differences. Furthermore, a larger sample size is needed when there is a larger variability in turning points. Finally, the location of turning point(s) has an effect on sample size. For any difference between turning points and for any variability in turning points, the smallest sample size is needed if the mean turning point is located halfway through the study (*μ*_*T*_ = 6). In the case where all subjects have the same turning point (zero ${\sigma}_T^2$), the required sample size for *μ*_*T*_ = 3 is equal to that for *μ*_*T*_ = 9. In the case where subjects have different turning points, the required sample size for *μ*_*T*_ = 3 is smaller than that for *μ*_*T*_ = 9.

**Table 1:** Sample size to achieve a power level 1 − *β* = 0.8 for the test on differential slopes

	*β*_1_ − *β*_2_ = 0.05			*β*_1_ − *β*_2_ = 0.16			*β*_1_ − *β*_2_ = 0.39		
	Zero ${\sigma}_T^2$	Small ${\sigma}_T^2$	Large ${\sigma}_T^2$	Zero ${\sigma}_T^2$	Small ${\sigma}_T^2$	Large ${\sigma}_T^2$	Zero ${\sigma}_T^2$	Small ${\sigma}_T^2$	Large ${\sigma}_T^2$
Early (*μ*_*T*_ = 3)	929	948	962	91	93	94	16	16	16
Halfway (*μ*_*T*_ = 6)	871	875	879	86	86	86	15	15	15
Late (*μ*_*T*_ = 9)	929	970	1003	91	95	98	16	16	17

Note: Two-sided test at type I error rate *α* = 0.05

**Fig. 3:**
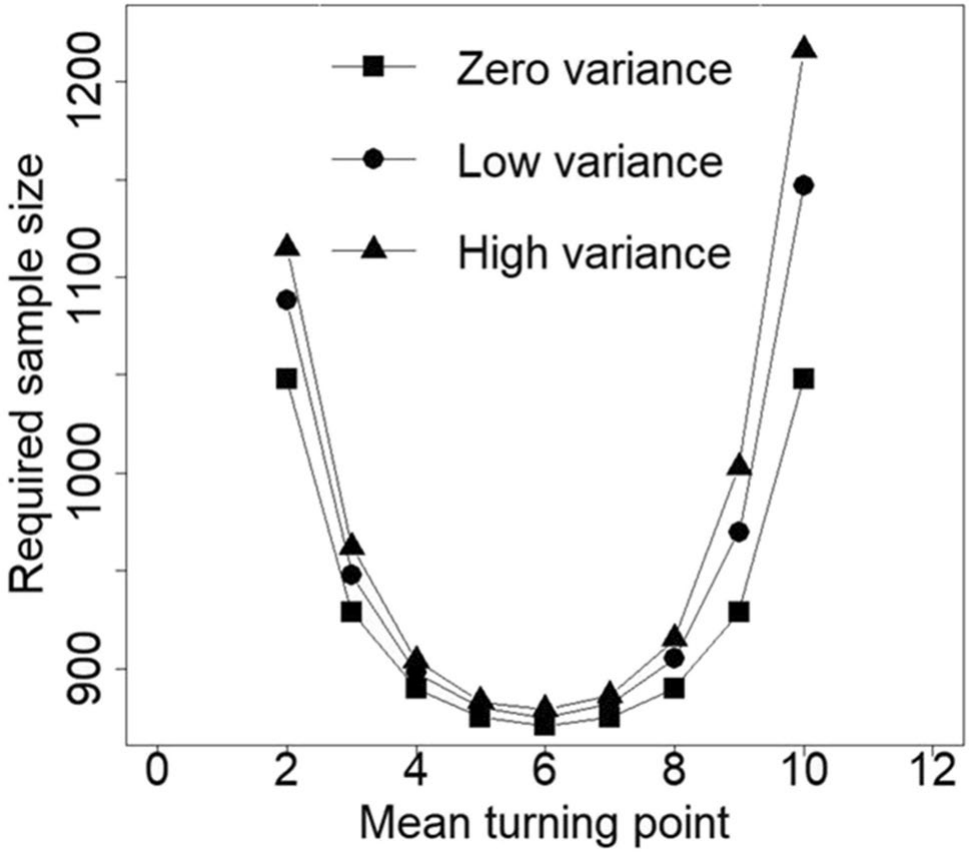
Required sample size to detect a difference in growth rates of *β*_1_ − *β*_2_ = 0.05 (1 − *β* = 0.8, *α* = 0.05, two-sided test) as a function of the distribution of turning points

The latter finding is further illustrated in Fig. 3, which shows the required sample size to detect a slope difference *β*_1_ − *β*_2_ = 0.05 as a function of the mean turning point and variability in turning points. The curve for the case of zero variability is symmetric around *μ*_*T*_ = 6, while the other two curves are not. In the case of between-subject variation in turning points, a larger sample size is needed for a late turning point than for an early turning point (having the same time difference from *μ*_*T*_ = 6).

## Designing studies with attrition

Attrition implies that subjects drop out during the course of a study. As a result, a larger sample size is needed to achieve a desired power level as compared to a study that is not hampered by attrition. This section investigates the increase in the required sample size due to attrition based on the Weibull survival function.

### Weibull survival function

**Fig. 4:**
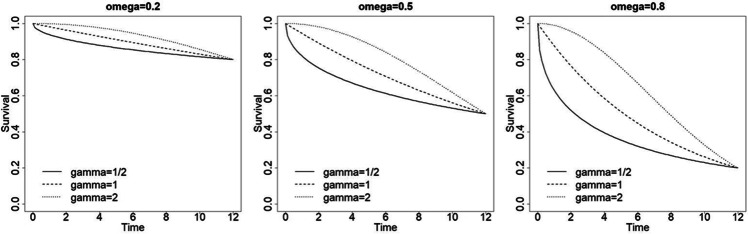
Weibull survival functions for various values of the survival probability *ω* and shape parameter *γ*

It is assumed that the underlying attrition process is continuous, meaning subjects may drop out at any time during the course of the study. Furthermore, it is assumed that attrition depends on the study time elapsed, but not on the number of measurements that are planned to be taken on each subject during the course of the study. The survival function gives the probability of staying in the study up to at least time point *t*: *S*(*t*) = *P*(*τ* > *t*), where *τ* is a continuous random variable measuring the elapsed study time. There exist many survival functions; in this paper the Weibull survival function is used. This is a flexible survival function in the sense that it allows for increasing, decreasing or constant attrition rates over time. The survival function is *S*(*t*) = exp(−*λt*^*γ*^). For the sake of convenience, time is rescaled by dividing by the study duration *D*, so that *t*_1_ = 0 is baseline and *t*_*m*_ = 1 is the last measurement. Furthermore, the parameter *λ* is replaced by − log(1 − *ω*), where *ω* ∈ [0, 1] is the proportion of subjects who drop out during the course of the study. The Weibull survival function is then formulated as $S(t)={\left(1-\omega \right)}^{t^{\gamma }}$. The parameter *γ* ∈ [0, ∞] determines the shape of the survival function. For *γ* < 1, the attrition rate decreases during the course of the study, meaning that attrition is concentrated at the beginning of the study. The opposite is the case for *γ* > 1, where the attrition rate increases during the course of the study, meaning that attrition is concentrated at the end of the study. A constant attrition rate is observed when *γ* = 1. Figure 4 shows survival functions for *ω* = 0.2, 0.5, 0.8 and for $\gamma =\frac{1}{2},1,2$.

To calculate the effect of attrition on the power to detect a difference in growth rates, the vector *N* = (*n*_1_, *n*_2_, …, *n*_*m*_)′, with *n*_*j*_ the number of subjects having *j* time points, needs to be known beforehand. However, this vector is random, with associated probability vector *p* = (*p*_1_, *p*_2_, …, *p*_*m*_)′, where *p*_*j*_ is the probability of having exactly *j* measurements. For each possible vector *N*, the variance in the estimator of the difference in growth rates across the two phases can be calculated. The expected variance is then the weighted variance across all possible vectors *N*, with the weights equal to the probability of each vector. This procedure becomes difficult to apply in studies where the number of time points, and hence the number of vectors *N*, is large. It is then useful to approximate the variance in the estimator of the difference in growth rates using a sampling procedure (Verbeke & Lesaffre, 1999). The vector *N* is sampled a large number of times using probability vector *p*, and for each draw the variance in the estimator of the difference in growth rates is calculated. The mean of these variances across all draws is then used to calculate the effect of attrition. A good approximation is made when the number of draws is large, which makes this procedure time-consuming. For that reason, a further approximation is made in this contribution. The vector *N* is replaced by its expectation *E*(*K*) = *n* × *p*. This procedure produces results similar to the sampling procedure (Galbraith & Marschner, 2002).

The variance in the treatment effect estimator is calculated based on Eq. (9). The following algorithm has been implemented in the Shiny app. First, given the distribution of turning points and sample size, calculate how many subjects there are for each turning point. Second, for each turning point, calculate the number of subjects with 1, 2, …, *D* measurement occasions. This number follows from the Weibull survival function with parameters *γ* and *ω*. Third, for each combination of turning point and number of measurements, construct the design matrix ***X***_*i*_ and covariance matrix of the random effects ***V***_*i*_ and calculate ${\boldsymbol{X}}_i^{\prime }{\left({\hat{\boldsymbol{V}}}_i\right)}^{-1}{\boldsymbol{X}}_i$. Fourth, multiply these terms by their associated sample sizes, sum up and take the inverse.

### Results

Table 2 shows the percentage increase in the required sample size to detect a difference in growth rates of *β*_1_ − *β*_2_ = 0.05 with a power level 1 − *β* = 0.8 in a two-sided test at type I error rate *α* = 0.05 as compared to a study without attrition. In the worst case, sample size needs to be increased by 259%, and in the best case by only 4%. As is obvious, a larger percentage increase is observed when more subjects drop out (i.e., larger *ω*) and when the risk of dropout is highest at the beginning of the study (i.e., larger *τ*). Furthermore, the largest percentage increase in sample size is observed when the turning points are located at the end of the study (larger *μ*_*T*_). This is obvious because, in that case, many subjects may have dropped out before their turning point. The variability in turning points, however, has a minor effect on the percentage increase in sample size.

**Table 2:** Percentage increase in sample size to achieve a power level 1 − *β* = 0.8 for the test on differential slopes as compared to a study without attrition

	Zero ${\sigma}_T^2$			Small ${\sigma}_T^2$			Large ${\sigma}_T^2$		
	$\tau =\frac{1}{2}$	*τ* = 1	*τ* = 2	$\tau =\frac{1}{2}$	*τ* = 1	*τ* = 2	$\tau =\frac{1}{2}$	*τ* = 1	*τ* = 2
	*ω* = 0.2								
Early (*μ*_*T*_ = 3)	14	9	4	14	9	4	14	9	4
Halfway (*μ*_*T*_ = 6)	16	12	7	16	12	7	16	11	7
Late (*μ*_*T*_ = 9)	18	16	13	18	15	13	18	15	12
	*ω* = 0.5								
Early (*μ*_*T*_ = 3)	49	29	13	49	28	13	48	28	13
Halfway (*μ*_*T*_ = 6)	59	42	25	59	42	25	58	41	25
Late (*μ*_*T*_ = 9)	70	59	48	70	59	46	70	58	46
	*ω* = 0.8								
Early (*μ*_*T*_ = 3)	152	78	31	150	77	31	149	76	31
Halfway (*μ*_*T*_ = 6)	201	132	70	200	130	69	199	128	68
Late (*μ*_*T*_ = 9)	259	214	166	258	210	157	256	206	150

Note: Two-sided test at type I error rate *α* = 0.05 and a difference in slopes of *β*_1_ − *β*_2_ = 0.05

## Example: alcohol use in middle and high school

Li et al. (2001) demonstrated the use of a piecewise growth model to study how alcohol use develops during middle (grades 6–8) and high school (grades 9–12). They used a mixture model to distinguish pupils with high (*N* = 57) and low (*N* = 122) initial status and developed piecewise models for both these groups. All subjects had the same turning point.

Suppose a replication of this study is to be conducted and an a priori sample size calculation is requested by the funding agency. The main research question is whether the growth rates during middle and high school differ from one another. Here it is illustrated how to calculate the required sample size for the high initial status group. Parameter estimates from Li et al. (2001) are used as input for the sample size calculation: ${\hat{\beta}}_0=2.594$, ${\hat{\beta}}_1=0.022$, ${\hat{\beta}}_2=0.255$, ${\hat{\sigma}}_{u0}^2=0.116$, ${\hat{\sigma}}_{u1}^2=0.092$, ${\hat{\sigma}}_{u2}^2=0.054$, ${\hat{\sigma}}_{01}=-0.031$, ${\hat{\sigma}}_{02}=-0.022$ and ${\hat{\sigma}}_{12}=-0.051$. Note that Li et al. (2001) analyzed their data using the latent growth curve model, which allows the residual variance *e*_*ti*_ to vary across the measurement occasions. The methodology for power analysis in this manuscript is based on the multilevel model, which is restricted to equal residual variance across time. For that reason, the mean of the estimates was used: $mean\left({\hat{\sigma}}_e^2\right)=0.23$.

**Fig. 5:**
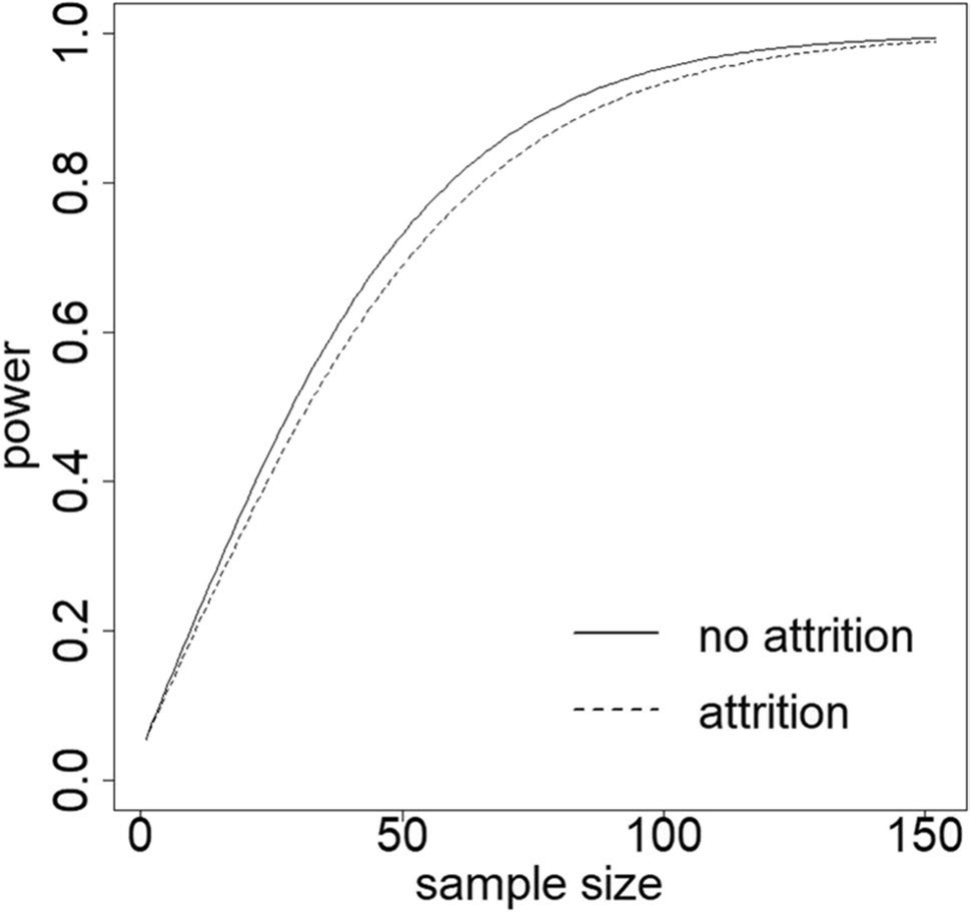
Power levels for the alcohol use example

Figure 5 shows the relation between sample size and power in the case where attrition is absent and when attrition is present and modeled by the Weibull survival function with *ω* = 0.25 and *γ* = 1 (meaning 25% of the students drop out during the study, and they do so at a constant rate). In the case where attrition is absent, a sample of size *N* = 60 should be used to detect a difference in growth rates at power 1 − *β* = 0.8, and type I error rate *α* = 0.05 in a two-sided test. This sample size is only slightly larger than the actual sample size used by Li et al. (2001). If attrition is present, then the required sample size increases a little further to *N* = 66.

## Conclusions and discussion

This study investigated the power to detect differential growth in linear–linear piecewise growth models. The relation between sample size and power was calculated for the multilevel mixed model. In a study without attrition, the required sample size is smallest when all subjects have the same turning point, which is located halfway through the study. Attrition increases the required sample size, especially when many subjects drop out and most of them do so at the beginning of the study.

A Shiny app was developed to facilitate the performance of a power analysis for future studies. To use the app, a priori estimates of the (co)variance components, residual variance and growth rates in both phases need to be specified. These can be obtained from the literature. In the example on alcohol use in middle and high school, parameter estimates from the literature were used. The required sample size turned out to be only slightly larger than that actually used by Li et al. (2001). However, that will not always be the case, and a power analysis is always preferred to basing the sample size for a future study on that of similar studies in the past. For that reason, it is important that estimates of (co)variance components, residual variance and growth rates in both phases are clearly reported in the literature, so that these can be used to calculate sample size for future studies.

Furthermore, the distribution of turning points needs to be specified a priori, along with the parameters *ω* and *γ* of the Weibull attrition function. In some studies the distribution of the turning points is under the control of the researcher. For instance, in psychotherapy trials, the therapy and follow-up phases may be of fixed duration, meaning that all participants have the same turning point and the location of the turning point is known beforehand. In trials in which a stepped-wedge design is used (Mdege et al., 2011), subjects move from the control to the intervention condition at preset points in time. In such trials there is variability in turning points but the number of turning points and the number of subjects that switch to the intervention at each turning point are under control of the experimenter and hence known beforehand. In observational studies, on the other hand, the distribution of turning points is often not known beforehand. In studies on developmental psychology, for instance, the turning point may be the transition from childhood to adolescence, and this turning point varies across subjects. However, the literature may provide good insight into the distribution of such a turning point. For studies in which no prior information about the distribution of turning points is available, the Shiny app may be used to explore the effects of various realistic distributions. The same applies, of course, to the parameters *ω* and *γ* of the Weibull attrition function.

This study extends previous work on power for piecewise growth models (Diallo & Morin, 2015; Segalas et al., 2019) by allowing for variability in turning points and non-constant attrition. Future extensions may focus on studies with more than one turning point (Cudeck & Harring, 2007; Harring et al., 2021; Marcoulides, 2018), studies with nonlinear growth in one or more phases (Flora, 2008; Harring et al., 2021; Zvoch, 2016), and studies with individually varying times of observation (Liu et al., 2015). It is also of interest to focus on models for discontinuous growth, meaning that there is not only a change in growth rate at the turning point, but also a change in level (Grimm & Marcoulides, 2016). Finally, it is worthwhile to focus on power analysis in the case of non-continuous outcome variables and to explore the effects of covariates on power.

In conclusion, this contribution presents power analysis for linear–linear piecewise growth models, taking into account the possibility of variability in turning points and non-constant attrition rates. I hope the results presented in this contribution, along with the Shiny app, will be helpful in calculating sample sizes for future research.

## Supplementary Information

ESM 1 (DOCX 18 kb)

## Data Availability

Data sharing is not applicable to this article as no datasets were generated or analyzed during the current study.
