# Supplementary material for: Power analysis of longitudinal studies with piecewise linear growth and attrition
Source: Behav Res Methods. 2022 Feb 7;54(6):2939–48. doi: 10.3758/s13428-022-01791-x (PMC9729151; doi:10.3758/s13428-022-01791-x)
Supplement: Supplementary file 1 — (DOCX 18 kb) [file 13428_2022_1791_MOESM1_ESM.docx]

Supplementary material to:

Power analysis of longitudinal studies with piecewise linear growth and attrition

A small simulation study was conducted to verify if the power levels obtained from matrix algebra are similar to those obtained from simulation.

The following population values of the regression coefficients were used

$\left( \beta_{0},\beta_{1},\beta_{2} \right)=(1,0.16,0.11)$.

The population value of the difference between both slopes is thus $\beta_{1}-\beta_{2}=0.05$. The following population values for the variance and covariance components were used:

$\left( \begin{matrix} \sigma_{u0}^{2} & \sigma_{01} & \sigma_{02} \\ \sigma_{01} & \sigma_{u1}^{2} & \sigma_{12} \\ \sigma_{02} & \sigma_{12} & \sigma_{u2}^{2} \end{matrix} \right)=\left( \begin{matrix} 0.2 & 0.1 & 0 \\ 0.1 & 0.1 & 0 \\ 0 & 0 & 0.16 \end{matrix} \right)$.

Finally, the population value of the residual variance was set at $var(e_{ij})=0.2$ and did not vary across the time points. The nine distributions of turning points as given in Figure 2 of the paper are used and for each of them sample sizes of $n=100$, $n=500$ and $n=1000$ are considered.

For each distribution of turning points and sample size, 5000 datasets were generated in R version 4.1.1. and subsequently analyzed using the lmer function with full information maximum likelihood estimation from the package lme4. The power was calculated as the number of simulated datasets for which the null hypothesis of equal slopes was rejected in a two-sided test at type I error rate $\alpha=0.05$.

The results are given in the table below. As can be seen, the power levels obtained from matrix algebra and those obtained from simulation are very similar.

Power levels for the test on differential slopes as obtained from on matrix algebra and a simulation study.

|  | Zero $\sigma_{T}^{2}$ | Small $\sigma_{T}^{2}$ | Large $\sigma_{T}^{2}$ |
| --- | --- | --- | --- |
|  | $n=100$ subjects | | |
| Early turning point ($\mu_{T}=3$) | 0.1491 [0.1594] | 0.1469 [0.1532] | 0.1453 [0.1604] |
| Halfway turning point ($\mu_{T}=6$) | 0.1561 [0.1574] | 0.1556 [0.1594] | 0.1552 [0.1612] |
| Late turning point ($\mu_{T}=9$) | 0.1491 [0.1664] | 0.1445 [0.1502] | 0.1412 [0.1396] |
|  | $n=500$ subjects | | |
| Early turning point ($\mu_{T}=3$) | 0.5383 [0.5512] | 0.5299 [0.5376] | 0.5239 [0.5464] |
| Halfway turning point ($\mu_{T}=6$) | 0.5648 [0.5722] | 0.5627 [0.5584] | 0.5612 [0.5682] |
| Late turning point ($\mu_{T}=9$) | 0.5383 [0.5572] | 0.5207 [0.5242] | 0.5075 [0.5100] |
|  | $n=1000$ subjects | | |
| Early turning point ($\mu_{T}=3$) | 0.8284 [0.8240] | 0.8207 [0.8252] | 0.8150 [0.8096] |
| Halfway turning point ($\mu_{T}=6$) | 0.8515 [0.8432] | 0.8497 [0.8424] | 0.8484 [0.8418] |
| Late turning point ($\mu_{T}=9$) | 0.8284 [0.8318] | 0.8120 [0.8150] | 0.7991 [0.8074] |

Note: two-sided test at type I error rate $\alpha=0.05$. The first entry within each cell is the power as obtained from matrix algebra; the second entry is the empirical power based on a simulation study.
